# “Publish or perish”: Should this still be true for your data?

**DOI:** 10.1016/j.dib.2014.11.005

**Published:** 2014-11-28

**Authors:** Hao-Ran Wang

**Affiliations:** Novartis Institutes for BioMedical Research, 250 Massachusetts Avenue, Cambridge, MA 02139, USA

The past decades have witnessed an era of data explosion that touches almost every aspects of modern science. Powerful technologies such as Next-gen DNA sequencing, high throughput/content screening platforms, the Large Hadron Collider and more, routinely generate copious amounts of data. At the same time, collaborations between different disciplines had never been so urgently needed to provide new insights that otherwise would not be possible. We are in need of a revolution in publication infrastructure towards supporting researchers with the means to preserve and gain access to research output. In this context, Elsevier has proudly launched *Data in Brief*, an open access journal that publishes data from a broad spectrum of disciplines including Biology, Chemistry, Economics, Psychology and Physics. *Data in Brief* strives to provide a platform for researchers to easily share and reuse each other׳s datasets, promote collaboration across disciplines and to showcase the benefits of data driven research.

I have personally experienced research in both academic and industrial settings. If I were asked to single out one thing that impressed me most about pharmaceutical industry, it will be the systematic approach the industry adopted for collecting research and clinical data. Bringing a drug to the market heavily relies on the data collected during the research and development process. Once data are generated in pharmaceutical institute, they are pooled into a centralized database together with very detailed lab note descriptions of the protocols, experimental designs, and analysis. The pharmaceutical industry has spared no efforts in centralizing data to facilitate decision-making as well as innovation. For example, we collect hundreds of assay data around a specific compound and place them in a single webpage to facilitate decision making on how to optimizing the lead compound. I can easily pull out a complete raw dataset for an experiment done 20 years ago as my reference in Novartis Institutes for BioMedical Research (NIBR).

The push for data sharing and archiving in academia is starting to pick up speed and could benefit from the lessons already learned in industry. It is still the case that data generated in an academic setting may sit in the lab for decades without being published or known to the outside world, waiting for a “story” that could never be completed. Personally, I still have a lot of interesting data from my Ph.D. study yet to be published, which could have provided insights for other researchers to work on. It is sad that “publish or perish” still holds true today when the information storage and sharing costs almost nothing. Those “perished data” indeed posed a tremendous waste of the valuable time and funding spent to collect them. It is my sincere hope that *Data in Brief* will provide a venue for researchers from academia and industry to not only share their data, but also share their unique experiences in data handling and archiving.

Moreover, we see opportunities for *Data in brief* to facilitate collaboration across disciplines. We do not assume we know what data can be useful to others, so *Data in Brief* welcomes submissions that describe data from all research areas. With this in mind, we deeply appreciate the diversity of data sources for *Data in brief* to incorporate, and allow great flexibility on the formality of the dataset to be accompanied with the body of data description, as long as they provide sufficient clarity for reproducibility and interpretation.

*Data in brief* currently publish papers from 1. Collaborating journals that encourage authors to convert their supporting data into a Data in Brief article; these articles are editorially reviewed; and 2. Directly submitted data articles which are sent for peer-review to verify the value and transparency of the data. To meet the speed of data generation, we aim to streamline the writing/reviewing process in order to minimize the barrier for authors to write up data articles while maximally preserving the fidelity of the data for the benefit of reproducibility and future interpretation. Authors are asked to create their data reports by following the downloadable *Data in brief* template. We ask for key information such as Specifications and Value of the data, Experimental Design, Supplemental data, etc. Peer reviewers for *Data in Brief* will verify that the articles provide sufficient details on how data were acquired, good reasons why the data are important, and that any base level analysis of the data is well described and relevant computer code provided. Data articles do not provide interpretation. In addition to original data articles submission, authors are very welcome to translate their research article׳s supporting data into a Data in Brief to be co-published with their research article. In addition to original data articles, we also consider (1) negative results data, (2) quality control data for evaluating commercial products, and (3) perspectives and commentary articles.

We hope *Data in brief* can be part of the solution to ease the publication barriers for excellent data, encourage data sharing and contribute to establishing community standards for data preservation and sharing.

